# Deep learning-based classification for lung opacities in chest x-ray radiographs through batch control and sensitivity regulation

**DOI:** 10.1038/s41598-022-22506-4

**Published:** 2022-10-20

**Authors:** I-Yun Chang, Teng-Yi Huang

**Affiliations:** Department of Electrical Engineering, National Taiwan University of Science and Technology, Taipei, Taiwan

**Keywords:** Medical research, Biomedical engineering

## Abstract

In this study, we implemented a system to classify lung opacities from frontal chest x-ray radiographs. We also proposed a training method to address the class imbalance problem presented in the dataset. We participated in the Radiological Society of America (RSNA) 2018 Pneumonia Detection Challenge and used the datasets provided by the RSNA for further research. Using convolutional neural networks, we implemented a training procedure termed batch control to manipulate the data distribution of positive and negative cases in each training batch. The batch control method regulated and stabilized the performance of the deep-learning models, allowing the adaptive sensitivity of the network models to meet the specific application. The convolutional neural network is practical for classifying lung opacities on chest x-ray radiographs. The batch control method is advantageous for sensitivity regulation and optimization for class-unbalanced datasets.

## Introduction

Pneumonia is an inflammatory condition of the lung that primarily affects the small air sacs called alveoli, and it is usually caused by bacterial or viral infection. Pneumonia symptoms typically include cough with phlegm, chest pain, difficulty breathing, and fever, and the severity of symptoms can vary. Pneumonia usually manifests as an area or areas of opacity on chest radiographs (CXRs). However, pneumonia diagnosis is complicated because increased opacity on CXRs could represent several other lung conditions, such as pulmonary edema, bleeding, volume loss, and lung cancer.

In recent years, deep-learning methods based on convolutional neural networks (CNNs) have exhibited increasing potential and efficiency in image recognition tasks, such as robotics^1^, self-driving cars^2,3^, and medical applications^4–7^. For application to CXR, deep-learning models can achieve detection performance close to that of radiologists^8,9^. Rajpurkar et al. developed CheXNet^10^ by using the ChestX-ray14 dataset^11^, which contains 112,120 frontal-view chest x-ray images individually labeled with 14 different pathologies, including pneumonia. They estimated and compared the performance of the model and four radiologists, revealing that the model exceeded the average performance of the radiologists on the pneumonia detection task. Hwang et al. applied a deep-learning method for chest radiograph diagnosis in the emergency department^8^, with their results indicating that the diagnostic performance of radiology residents using CXR readings improved after radiographs were reinterpreted through the deep-learning algorithm output.

In the 2018 Radiological Society of America (RSNA) Pneumonia Detection Challenge (https://www.kaggle.com/c/rsna-pneumonia-detection-challenge), 1499 teams participated, 344 teams entered the second stage, and our team “TigerDuck” finished in 22nd place. The short competition period (August 28 to October 31, 2018) did not allow for a thorough performance assessment of this application. After the competition, we further investigated the dataset and CXR models and observed that class imbalance played a major role in the optimization of classification performance. In this study, we investigated the effect of imbalance classification in the CXR deep-learning model and proposed a batch control method (BCM) to regulate the sensitivity of deep learning–based CXR models.

## Methods and materials

### Datasets

**Figure 1:**
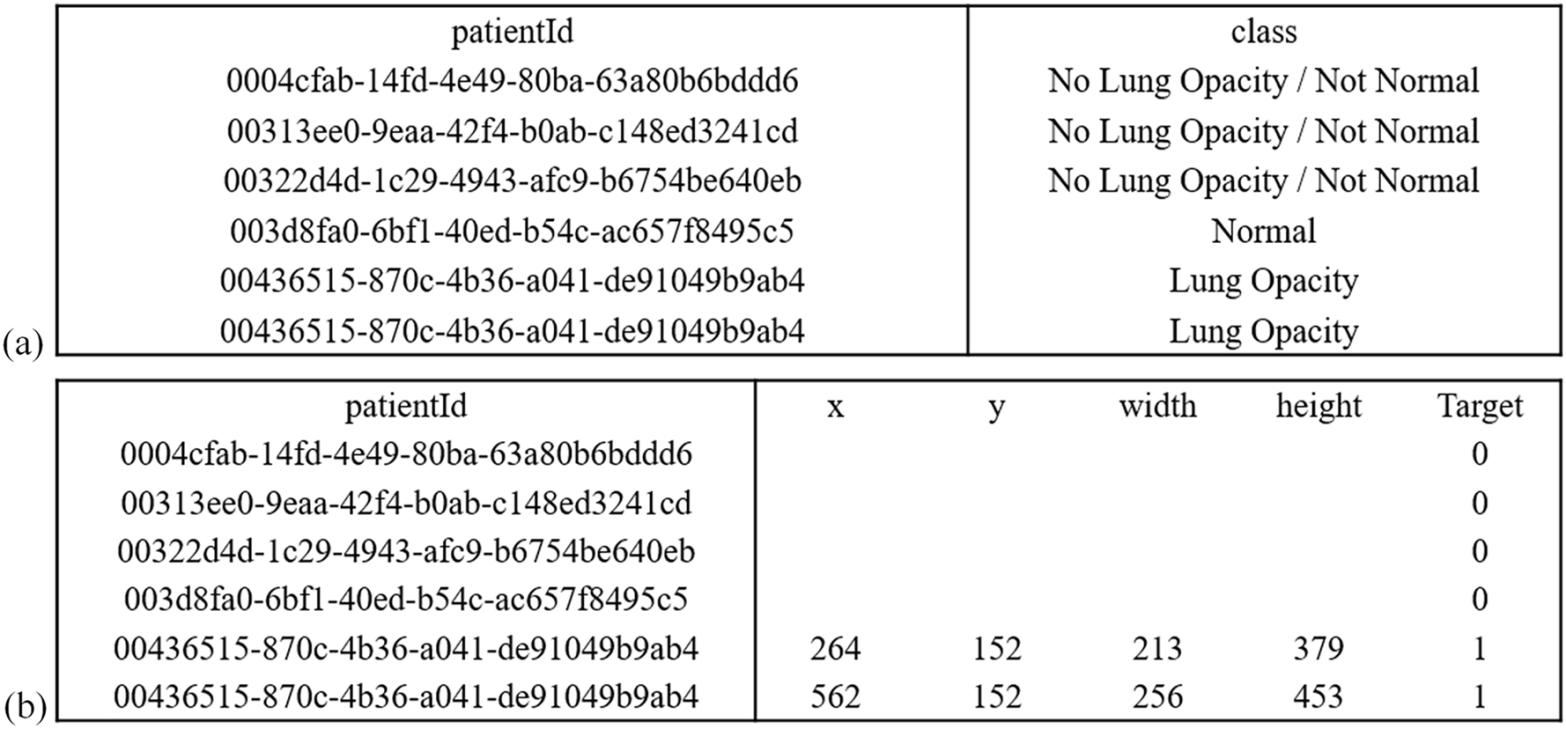
Five example images in the RSNA dataset. Details of (**a**) class information and (**b**) corresponding lung-opacity region. The coordinates, width, and height of a bounding box for each region.

**Figure 2:**
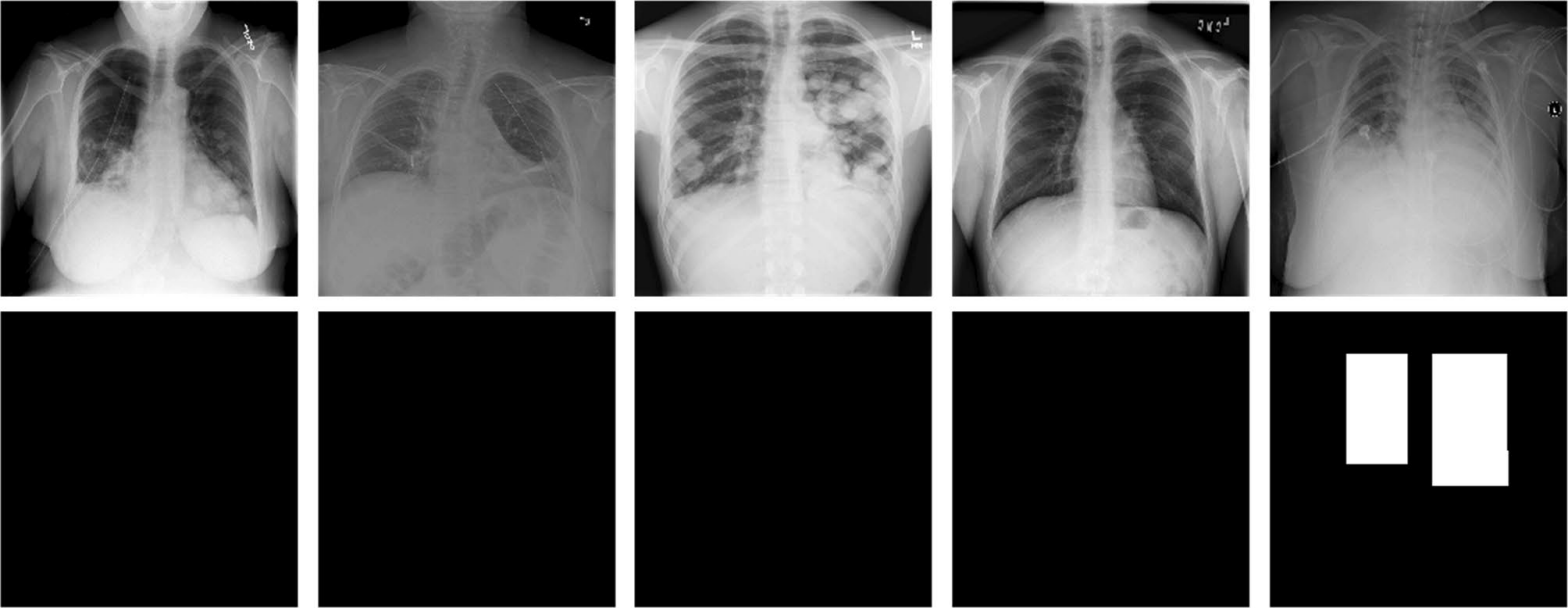
Images and labels corresponding to the five examples introduced in the Fig. 1.

The dataset used for model development and evaluation was obtained from the RSNA Pneumonia Detection Challenge and is part of the ChestX-ray14 dataset, which is publicly available from National Institutes of Health Clinical Center. The dataset consists of the class information, 25,684 frontal-view chest radiographs in Digital Imaging and Communications in Medicine (DICOM) format, and the bounding box information of corresponding labels of different patients. Table 1 lists the demographic characteristics of this dataset, and Fig. 1 presents five examples with class and bounding box descriptions. Figure 2 depicts the CXR images and the corresponding labels of the lung-opacity regions. All frontal chest radiographs were annotated by six board-certified radiologists. Three types of classes were identified in a total of 25,684 images, namely lung opacity (5659 images), normal (8525 images), and no lung opacity or not normal (11,500 images). We randomly selected 24,684 images to form a training set and 458 images for a testing set (lung opacity: 229 images; no lung opacity: 229 images). The data distribution is detailed in Table 1.

**Table 1:** Demographics of the training and test datasets.

Characteristic	Training set	Test set
Median age	49 (1 to > 90)	47.5 (1 to > 90)
**Gender**		
Male	14,033	261
Female	10,651	197
No. of images	24,684	458
AP images	11,298	239
PA images	13,386	219
**Data type**		
Lung opacity	5430	229
Normal	8194	114
No opacity/not normal	11,060	115

### Deep learning: semantic segmentation and UNet

**Figure 3:**
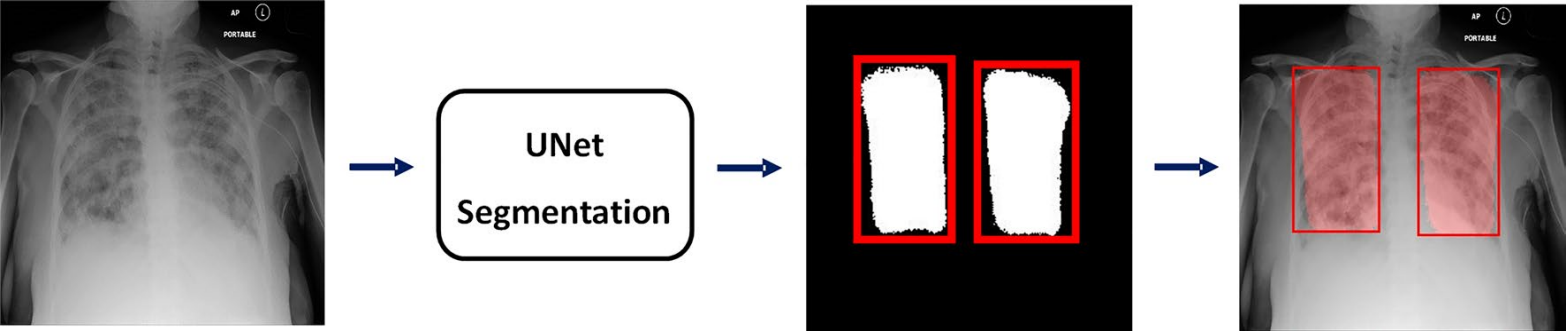
Block diagram of the CXR model based on UNet segmentation. After model training using UNet, we predicted the region of lung opacity pixel by pixel and then outlined the bounding boxes of the regions according to the segmentation boundaries.

In this study, we applied semantic segmentation to detect and locate the lung opacities of pneumonia in frontal CXR images (Fig. 3). Semantic segmentation refers to a process of pixel-wise classification of an image, where the boundary and location of target objects are learned pixel by pixel. The general architecture of semantic segmentation can be considered an encoder network followed by a decoder network. The encoder is generally a classification network, which extracts particular features from input images. After several encoder and decoder stages, each pixel of an input image is classified into a specific category.

In our study, the two-dimensional UNet was used for our semantic segmentation approach^12^. The UNet architecture consists of a contracting path to capture context on the left and an expanding path for precise localization on the right. The contracting path comprises stacks of 3 × 3 convolutional layers; each layer is followed by a rectified linear unit (ReLU function) and a 2 × 2 max-pooling operation with stride = 2 for downsampling. During the enconding stage, the number of CNN filters doubled at each downsampling stage. The expanding path contains the following: (1) an upsampling process of the feature map followed by 2 × 2 upconvolution, which halves the number of feature channels; (2) a concatenation with the corresponding feature map from the contracting path; and (3) two 3 × 3 convolutional layers followed by the ReLU function. Finally, a fully connected layer maps each component feature to the desired classes.

### BCM

For machine learning methods, gradient descent is the most widely used algorithm for optimizing CNNs. A batch refers to a group of training samples used in gradient-descent optimization, and depending on the number of samples (i.e., the batch size) in each training step, the following three modes of optimization can be performed: (1) batch mode, where the batch size is equal to the sample size; (2) stochastic mode, where only one sample is used in each training step; and (3) minibatch mode, where the batch size is between the batch and stochastic mode. Batch-mode learning uses the whole training set to obtain a loss value at each training step, consuming considerable computation resources for large datasets. Therefore, minibatch mode learning is generally used for deep-learning applications.

For minibatch gradient-descent optimization, we calculated the sum of gradients with respect to the samples in the minibatch and updated the network parameters by using this cumulative gradient. In general, a minibatch is randomly sampled from the whole dataset, and thus, the distribution of data classes varies in each batch, potentially leading to stochasticity of the obtained model. In this study, we proposed a BCM to manipulate the class distribution of a batch. Using a batch size of six, we altered the number of positive and negative cases in the lung-opacity classification model. Seven schemes of BCMs were used, denoted as P100, P83, P66, P50, P33, P17, and RAND. The corresponding proportions of positive samples were 100%, 83%, 66%, 50%, 33%, 17% and 22%, respectively. For example, a P100 batch contained six positive cases that were randomly selected from the training dataset, whereas a P83 batch contained five positive cases and one negative case. A RAND batch contained six randomly selected samples.

### Training, prediction, and evaluation

We trained the proposed UNet with the following parameters: optimizer, Adam; batch size, 6; batch mode, P100, P83, P66, P50, P33, P17, and RAND; loss function, cross-entropy, training steps, 60,000; and image augmentation, random image flipping, rotation angle (15° to − 15°), and contrast adjustments (0.6–1.4). We implemented the UNet structure with a TensorFlow framework (v1.8) and Python (v3.6) environment and trained the model on a personal computer with a graphics processing unit (GPU) (1080 Ti, Nvidia, Santa Clara, CA, USA). We performed eight trials for each data type combination and evaluated the performance of the models with the true-positive rate (TPR; also called sensitivity or recall), the true negative rate (TNR; also called specificity), accuracy (ACC), the false positive rate (FPR), F1-score, and the coefficient of variation of the F1-score (F1-CV).

## Results

**Figure 4:**
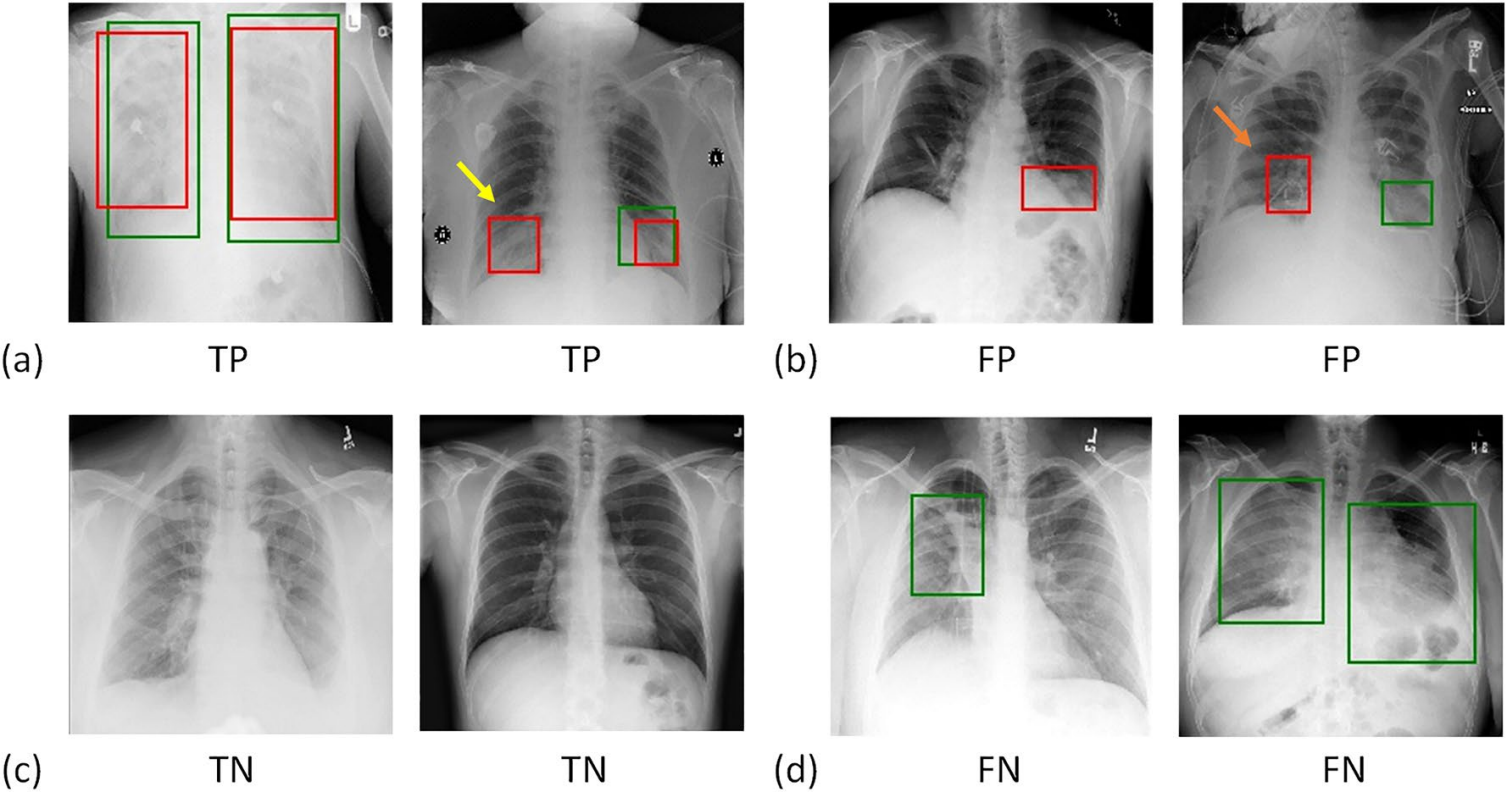
Example results of predicted cases, including (**a**) true positives (TP), (**b**) false positives (FP), (**c**) true negatives (TN), and (**d**) false negatives (FN). The true and predicted lung-opacity regions are denoted by green and red bounding boxes, respectively. Within the definitions of this study, we considered the second example in (**a**) to be a TP despite one of the two predicted boxes (yellow arrow) not overlapping with the reference box. We regarded the second example in (**b**) as an FP. Although the CXR model correctly predicted this as positive, the predicted region, indicated by the orange arrow, does not overlap with the green box.

Figure 4 illustrates examples of the predicted results from the CXR test set. Figures 4a and b demonstrate positive and negative predictions, respectively. The region of lung opacity of true and predicted labels are denoted by the green and red bounding boxes, respectively. In Fig. 4a, all CXR images exhibit red boxes, indicating classification as positive cases by the UNet model. The first two cases were classified as true-positive (TP) observations, and the last two cases were identified as false positive (FP) observations. Only when the green and red boxes overlapped did we designate a TP observation. For the second case, the predicted and true findings were both positive, and the green and red boxes overlapped. Although the model also generated an FP red box (indicated by the yellow arrow), we classified this prediction as a TP case. For the fourth case, although the predicted and true findings were both positive, we defined it an FP case because the predicted region (indicated by the orange arrow) did not overlap with the true region of lung opacity.

**Figure 5:**
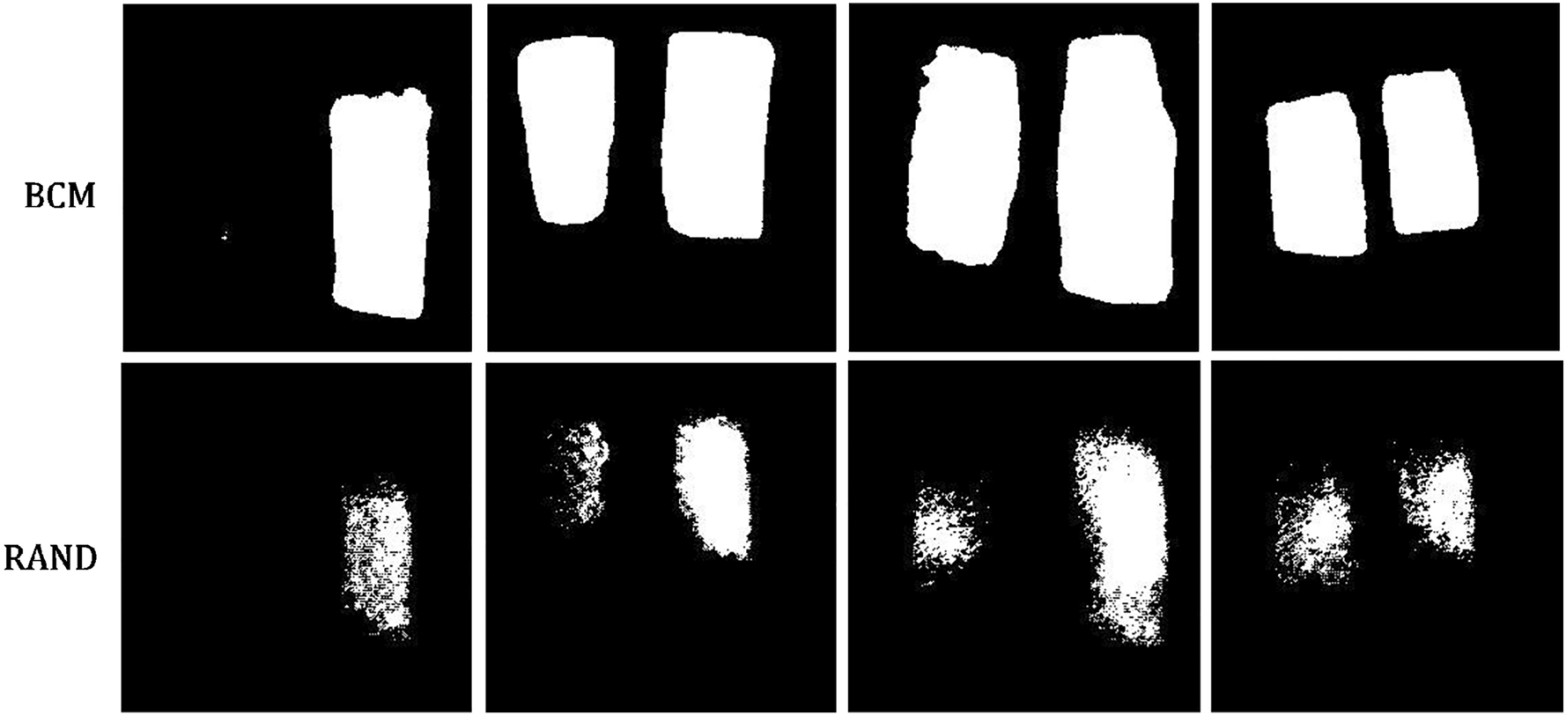
Example masks generated using the batch control method and random (RAND) models. The selected examples demonstrate the scattered pixels observable in some results generated from the RAND model.

**Figure 6:**
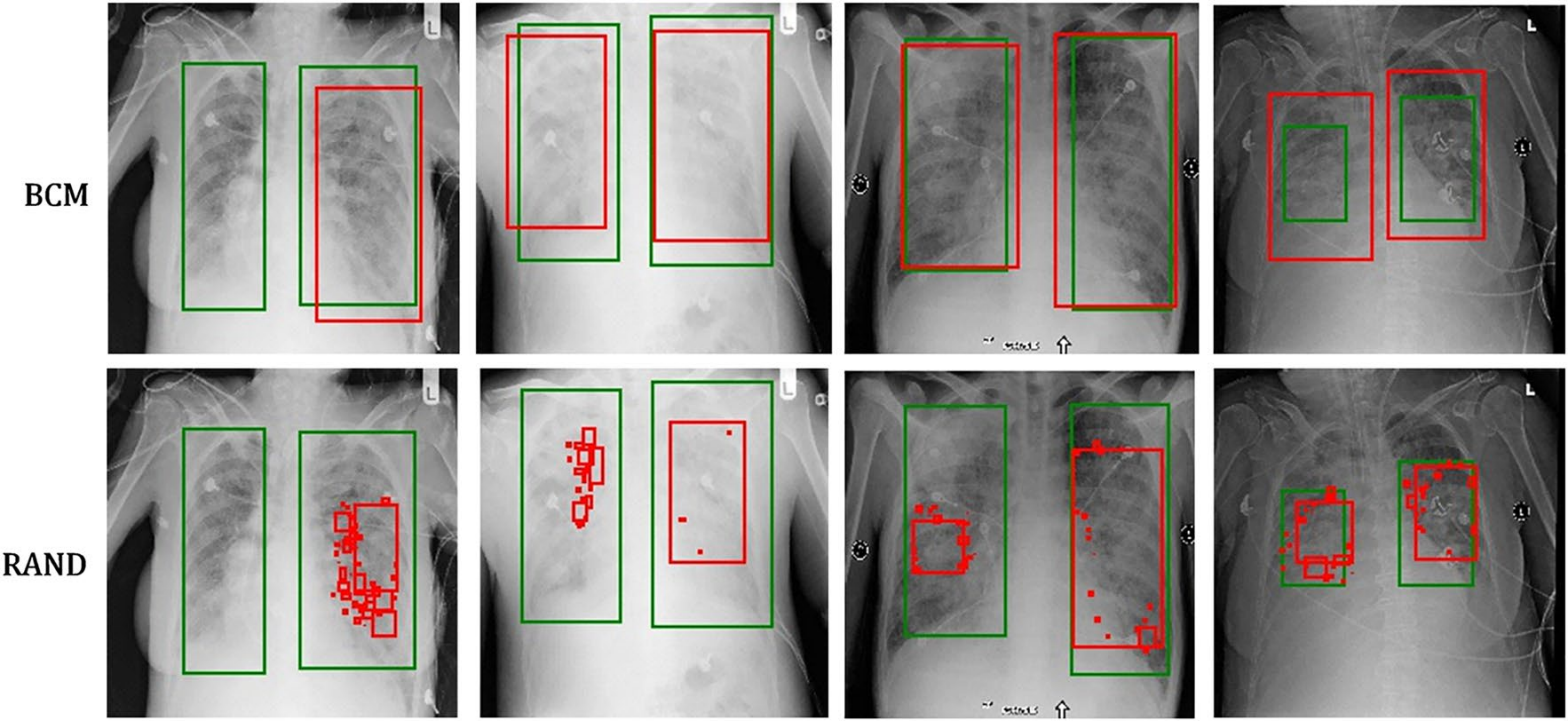
Bounding boxes generated from the batch control method and random models, corresponding to the masks in Fig. 5.

Table 2 lists the quantitative assessment of the CXR models obtained using the seven batch modes. From the P100 to P17 models, the number of positive cases decreased, the average TPR decreased, and the average TNR increased. The P83 model produced the highest F1-score (TPR: 0.80, TNR: 0.75, F1-score: 0.78), whereas the RAND model exhibited low performance (TPR: 0.46, TNR: 0.98, F1-score: 0.62). The F1-CV values (eight trials) of the BCM models ranged from 0.011 to 0.043, and the F1-CV of the RAND model was 0.135. The relatively low F1-CV of the BCM models suggested more stable results than those of the RAND model. In addition to the classification results, we discovered that the segmentation of the lung-opacity regions produced by the RAND model was sometimes tattered, leading to scattered localization of the predicted bounding boxes. Figure 5 presents the predicted results of four samples obtained using the BCM (upper row) and RAND (lower row) models, and Fig. 6 depicts the generated bounding boxes corresponding to Fig. 5. Scattered bounding boxes were visible in the area of true labels in the RAND results. The BCM results obtained from the same images exhibited largely intact bounding boxes. However, the prediction results of the BCM models revealed no such conditions, and the prediction areas were largely intact. The prediction results of BCM presented more complete shapes than those of the RAND model, which exhibited blurred, unclear contours with scattered pixels.

**Table 2:** Performance metrics for different CXR models.

Model	TPR	TNR	FPR	ACC	F1-score	F1-CV
P100	0.93 ± 0.01	0.23 ± 0.05	0.77 ± 0.05	0.58 ± 0.02	0.69 ± 0.01	0.019
P83	0.80 ± 0.03	0.75 ± 0.03	0.25 ± 0.03	0.78 ± 0.01	0.78 ± 0.01	0.012
P66	0.73 ± 0.05	0.83 ± 0.05	0.17 ± 0.05	0.78 ± 0.01	0.77 ± 0.02	0.024
P50	0.67 ± 0.03	0.88 ± 0.03	0.12 ± 0.03	0.78 ± 0.01	0.75 ± 0.01	0.011
P33	0.60 ± 0.04	0.93 ± 0.03	0.07 ± 0.03	0.77 ± 0.01	0.72 ± 0.02	0.033
P17	0.44 ± 0.03	0.99 ± 0.00	0.01 ± 0.00	0.71 ± 0.01	0.60 ± 0.03	0.043
RAND	0.46 ± 0.09	0.98 ± 0.01	0.02 ± 0.01	0.72 ± 0.04	0.62 ± 0.08	0.135

**Figure 7:**
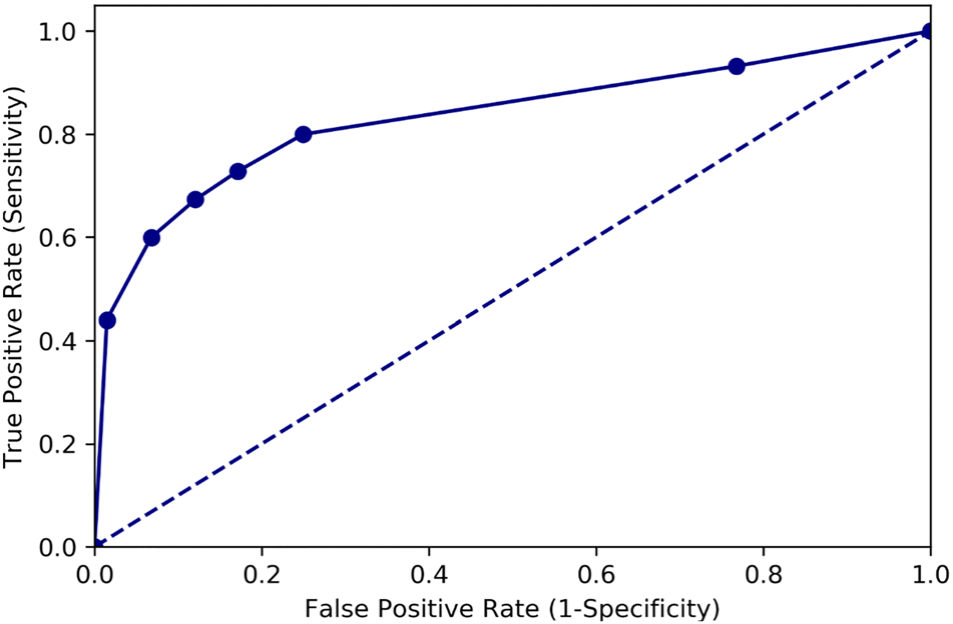
Analogous receiver operating characteristic (ROC) analysis of the UNet models obtained using batch control methods. The estimated area under the ROC curve is 0.83.

Figure 7 illustrates the TPR values plotted against those of FPR for the six BCM models; the relative trade-offs between TPR and FPR performance exhibited similarity to a receiver operating characteristic (ROC) analysis of a binary classifier system with varied thresholds for discrimination. The area under the ROC curve (AUC) provided a joint metric of the prediction performance despite classification thresholds. We linearly interpolated the six TPR–FPR pairs of the BCM models and calculated an analogous AUC value of the UNet CXR model. This value of 0.82 represented the model performance irrespective of which batch mode was chosen.

**Figure 8:**
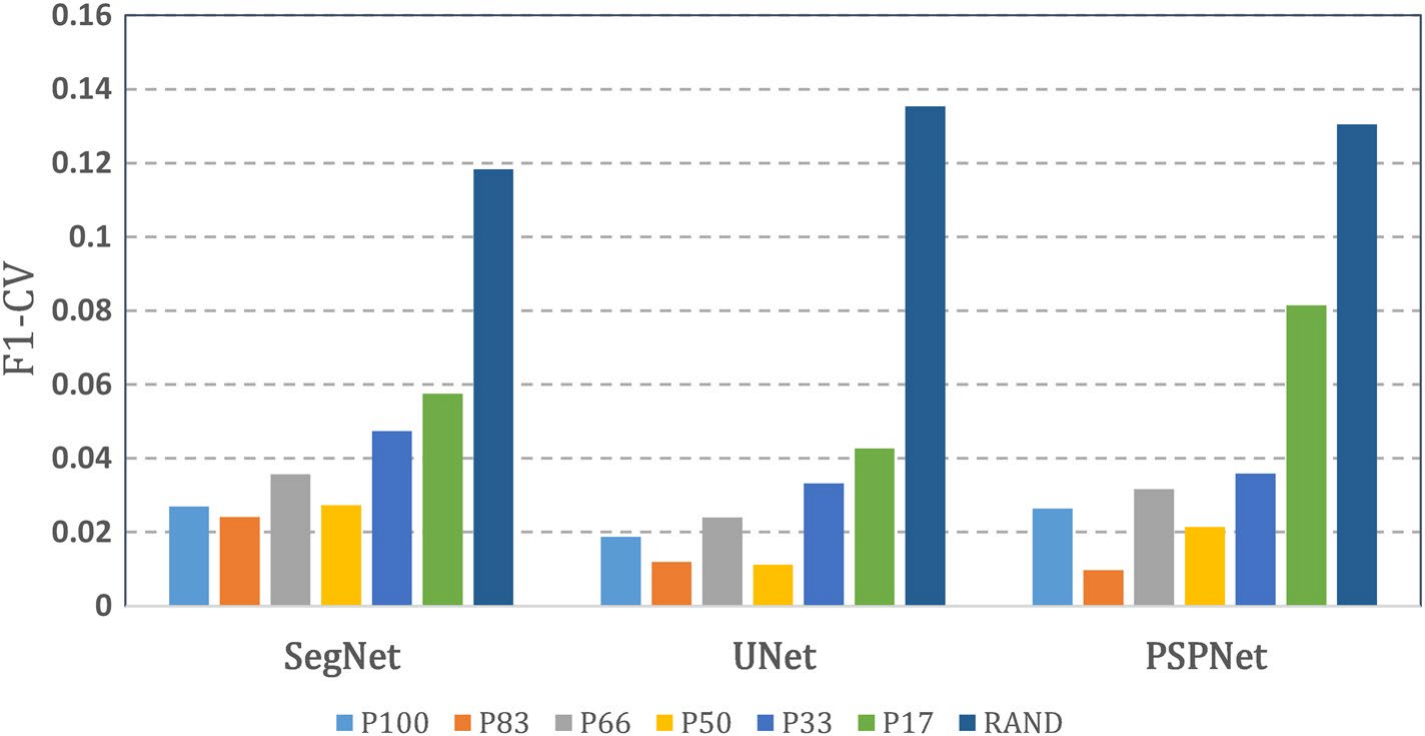
F1-CV values of the seven models obtained by SegNet, UNet, and PSPNet.

In addition to UNet, we used SegNet^13^ and PSPNet^14^ to form a semantic segmentation model. SegNet and PSPNet are widely used methods of semantic segmentation implementation. Overall, the model performance did not vary significantly across the three networks. As with the UNet results, we observed consistent TPR and TNR trends in the six batch modes (data not shown). However, the coefficient of variations in the F1-score identified differences. Figure 8 presents a comparison of the CVs of the F1-score between SegNet, UNet, and PSPNet with the seven batch modes. For the BCM models, the average F1-CV values obtained using SegNet, UNet, and PSPNet were 0.0363, 0.0236, and 0.0345, respectively. For the RAND model, the F1-CV values for the three nets were 0.095, 0.124, and 0.105, respectively. Regardless of the network structure, all BCM models exhibited prominently lower F1-CV scores than the RAND model did. Among the three networks, UNet had the lowest F1-CV values.

## Discussion

Imbalanced classification refers to the unequal distribution of data categories in the training dataset of a classification model and is a prevalent problem in data-driven deep-learning models. For example, the COVID-19 outbreak spread worldwide in mere months, affecting the lives and health of countless people. At the beginning of the outbreak, the CXRs of patients with confirmed cases of COVID-19 were available, but attempts to structure a deep-learning CXR model identifying COVID-19 cases with the unbalanced dataset would impede accurate performance assessment of the obtained model. In this study, we investigated BCM as a potential solution to imbalanced classification. The main methodological concept is the regulation of CXR model sensitivity by manipulating the data distribution of the data batches used for the training procedures.

In the dataset, the ratio between positive and negative cases was unbalanced (approximately 1:4). At the beginning of this study, we implemented the UNet model and trained it using a vanilla approach; we shuffled and created random batches of the dataset, fed these batches into the UNet model, and calculated the loss function for minibatch gradient-descent optimization. However, the result obtained in the preliminary stage was unsatisfactory and unstable. We then tailored the BCM to address the class imbalance problem. The BCM manipulates the distribution of positive and negative cases in each batch. For a batch size of six, we altered the number of positive cases from six to one and the number of negative cases from zero to five in each batch to create the P100 to P17 models. The vanilla UNet approach trained with a random data distribution produced the RAND model.

Following the class balanced test set (positive: 229, negative: 229 cases) and eight trials of training procedures, the P83 model (F1: 0.78) outperformed the other six models (F1: 0.62–0.77) in terms of F1-score. The other metrics of the P83 and RAND models were TPR = 0.80, FPR = 0.25, ACC = 0.78, and F1-CV = 0.012 for P83 and TPR = 0.46, FPR = 0.02, ACC = 0.72, and F1-CV = 0.135 for RAND. The F1-score of 0.62 for the RAND (the proportion of positive samples: 22%) approach was between those of the P33 (0.72) and P17 (0.60) models. The results indicate that the F1-scores were associated with the data distribution of training batches. In the three models (P33, P17, RAND), more negative cases were used in each iteration of network optimization. We anticipated that the networks would use a greater proportion of negative samples, which tended to result in more negative predictions. The TPR and FPR values from P100 to P17 were 0.93–0.44 and 0.77 to 0.01, respectively, which validate the association between data distribution and model performance. The results of P83, P66, and P50 presented higher F1-scores (0.75–0.78) than the other model did (0.60–0.72), indicating that the CXR model performed better when more than half of the training batch comprised positive samples. We can regulate the sensitivity of the CXR models by using the BCM to meet the requirements of different clinical environments. For example, if the identification of lung opacities in patients is a primary reason for CXR examination, a P83 BCM model may be suitable.

Across the eight trials of the training–testing procedures, the F1-CV values of the BCM models ranged from 0.011 to 0.043, and those of the RAND model ranged from 0.118 to 0.135, demonstrating that the BCM produced more stable results than did the RAND method. A fixed ratio of positive and negative samples resulted in a smoother loss function and led to better convergence in the BCM models. In our study, we investigated three networks: UNet, SegNet, and PSPNet; most of their performance metrics (e.g., TPR, and FPR) were similar except for the F1-CV. The average F1-CV values of the BCM models were 0.0236 (UNet), 0.0363 (SegNet), and 0.0345 (PSPNet), suggesting that the UNet method was the most stable.

In this study, a batch size of six was used because of the random access memory limit of the GPU (11 gigabytes in the GTX 1080Ti GPU card); therefore, the data distribution of the batch was limited to six variations, P100 to P17. With more GPU RAM, we may further improve the classification performance through the precise adaption of data distributions. For example, the result of P83 (positive:negative, P:N = 5:1) exhibited the best F1-score among the BCM models. We could produce P92 (P:N = 11:1) or P90 (P:N = 9:1) models to further optimize the CXR models. Supplement Fig. 1 displays our preliminary investigations on different batch sizes (batch size = 6, 9, 12, 18) and data ratios (P33, P66, P100). We can regulate the sensitivity of the CXR models by different batch sizes. Increasing the GPU RAM and the combinations in each batch merits further investigation.

In the machine learning methods, the learning procedures are generally biased towards the majority class because the classifiers aim to reduce global loss function. Therefore, the obtained model tends to misclassify the minority class in the dataset. To deal with the class imbalance problems, there exists several approaches in the machine learning field. At the data level, resampling methods, such as over-sampling, under-sampling or SMOTE^15–17^, generate a new dataset with adjusted data distribution. At the algorithm level, advanced loss functions, such as class rectification loss^18^ and focal loss^19^, taking the data distribution into loss derivation have been shown effective in the class imbalance problems. The BCM method proposed in this study can be considered as an implementation of the over-sampling method. The BCM method adjusts the proportion of positive samples in a batch and manipulates the over-sampling ratio during the training process. Combining the BCM method with advance loss functions may produce improved performance of the CXR models. Supplement Table 1 lists our preliminary comparison of cross-entropy loss and focal loss. The results suggested that RAND models trained with focal loss prominently improved the metrics of CXR classification. Future studies are necessary to validate the efficacy of the combined methods.

We used the segmentation network UNet for the classification application. The UNet architecture was used for the implementation of an encoder–decoder network with skip connections. In the field of deep learning–based pattern recognition, image classification can be also achieved with the encoder alone, followed by a fully connected output such as VGG16^20^, ResNet^21^, or DenseNet^22^. In addition, object-detection networks such as Faster Region-based CNN (R-CNN)^23^, YOLO^24^, or Mask R-CNN^25^ can be applied to CXR classification problems. We have not implemented or evaluated the BCM with these network architectures; this drawback is a limitation of this study. Nonetheless, we expect the BCMs to be advantageous because they are all based on convolutional networks. Further investigation of this theory is warranted.

In conclusion, we presented the deep-learning method as employed in the RSNA challenge for CXR recognition. To address the class imbalance of the RSNA dataset, we developed and evaluated the BCM. The models obtained using the BCM were more stable, and the sensitivity was adjustable through the manipulation of the distribution of positive and negative cases. Therefore, BCM is a practical method of producing regulable and stable CXR models regardless of whether the training dataset is imbalanced. The rapidly increasing number of confirmed COVID-19 infections continues to exert pressure on medical care systems and exhaust medical resources. As medical science researchers, we believe that global collaborative and investigative efforts will assist in overcoming this catastrophe.

## Supplementary Information

Supplementary Information.

## Data Availability

The datasets analyzed during the current study are available in the 2018 Radiological Society of America (RSNA) Pneumonia Detection Challenge, [https://www.kaggle.com/c/rsna-pneumonia-detection-challenge].
