# Supplementary material for: Deep learning-based classification for lung opacities in chest x-ray radiographs through batch control and sensitivity regulation
Source: Sci Rep. 2022 Oct 20;12:17597. doi: 10.1038/s41598-022-22506-4 (PMC9584230; doi:10.1038/s41598-022-22506-4)
Supplement: Supplementary file 1 — Supplementary Information. [file 41598_2022_22506_MOESM1_ESM.pdf]

SUPPLEMENT 1: BCM with different batch sizes

We trained the proposed UNet with the following parameters: optimizer, Adam; batch size, 6, 9, 12, and 18; data ratio, P100, P66, and P33; loss function, cross-entropy, training epoch, 10; GPU, Tesla V100 32G (Nvidia, Santa Clara, CA, USA). We performed four trials for each data type combination and evaluated the performance of the models with the sensitivity and F1-score. The performance metrics obtained using different batch sizes (9, 12, 18) showed a similar trend to the results produced by batch size 6. The results supported that the BCM models obtained with different batch sizes produced regulable and stable CXR models.

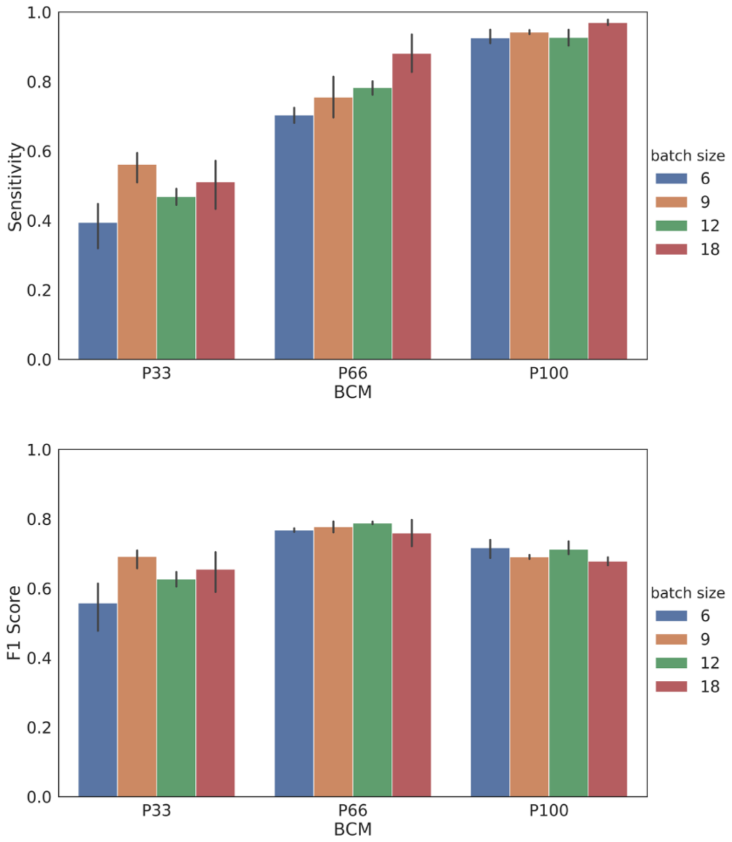

(a) (b)

Figure 1 BCM with different batch sizes. (a) Sensitivity (b) F1-score

SUPPLEMENT 2: Preliminary study on focal loss

We trained the proposed UNet with the following parameters: optimizer, Adam; batch size, 6; RAND batch; loss function, cross-entropy or focal loss (α=0.25, γ=1 or 2), training steps, 12000; GPU, Tesla V100 32G (Nvidia, Santa Clara, CA, USA). We performed four trials for each loss function and evaluated the performance of the models. Table 1 lists the performance metrics. The results suggested that focal loss (γ=1) prominently improved TPR and F1-score of the CXR classification model.

Table 1: Comparison of cross-entropy loss and focal loss

Loss	TPR	TNR	FPR	ACC	F1-score	F1-CV
Cross-Entropy	0.58±0.05	0.89±0.02	0.11±0.02	0.73±0.02	0.68±0.03	0.049
Focal (γ=1)	0.70±0.08	0.78±0.15	0.22±0.15	0.74±0.03	0.73±0.01	0.016
Focal (γ=2)	0.67±0.11	0.77±0.15	0.23±0.15	0.72±0.02	0.70±0.02	0.034
